# Rewiring glycerol metabolism for enhanced production of poly-γ-glutamic acid in *Bacillus licheniformis*

**DOI:** 10.1186/s13068-018-1311-9

**Published:** 2018-11-09

**Authors:** Yangyang Zhan, Bojie Sheng, Huan Wang, Jiao Shi, Dongbo Cai, Li Yi, Shihui Yang, Zhiyou Wen, Xin Ma, Shouwen Chen

**Affiliations:** 1 State Key Laboratory of Biocatalysis and Enzyme Engineering, Hubei Collaborative Innovation Center for Green Transformation of Bio-Resources, Environmental Microbial Technology Center of Hubei Province, College of Life Sciences, Hubei University, 368 Youyi Avenue, Wuhan, 430062 Hubei People’s Republic of China; 2 State Key Laboratory of Agricultural Microbiology, College of Life Science and Technology, Huazhong Agricultural University, Wuhan, 430070 People’s Republic of China; 3 College of Food Science and Technology, Huazhong Agricultural University, Wuhan, 430070 People’s Republic of China; 4 Department of Food Science and Human Nutrition, Iowa State University, Ames, IA 50011 USA

**Keywords:** *Bacillus licheniformis*, Poly-γ-glutamic acid, Glycerol metabolism, Gluconeogenesis pathway, NADPH, Combinatorial optimization

## Abstract

**Background:**

Poly-γ-glutamic acid (γ-PGA) is a natural polymer with great potential applications in areas of agriculture, industry, and pharmaceutical. The biodiesel-derived glycerol can be used as an attractive feedstock for γ-PGA production due to its availability and low price; however, insufficient production of γ-PGA from glycerol is limitation.

**Results:**

The metabolic pathway of *Bacillus licheniformis* WX-02 was rewired to improve the efficiency of glycerol assimilation and the supply of NADPH for γ-PGA synthesis. GlpK, GlpX, Zwf, and Tkt1 were found to be the key enzymes for γ-PGA synthesis using glycerol as a feedstock. Through combinational expression of these key enzymes, the γ-PGA titer increased to 19.20 ± 1.57 g/L, which was 1.50-fold of that of the wild-type strain. Then, we studied the flux distributions, gene expression, and intracellular metabolites in WX-02 and the recombinant strain BC4 (over-expression of the above quadruple enzymes). Our results indicated that over-expression of the quadruple enzymes redistributed metabolic flux to γ-PGA synthesis. Furthermore, using crude glycerol as carbon source, the BC4 strain showed a high productivity of 0.38 g/L/h, and produced 18.41 g/L γ-PGA, with a high yield of 0.46 g γ-PGA/g glycerol.

**Conclusions:**

The approach to rewiring of metabolic pathways enables *B. licheniformis* to efficiently synthesize γ-PGA from glycerol. The γ-PGA productivity reported in this work is the highest obtained in glutamate-free medium. The present study demonstrates that the recombinant *B. licheniformis* strain shows significant potential to produce valuable compounds from crude glycerol.

**Electronic supplementary material:**

The online version of this article (10.1186/s13068-018-1311-9) contains supplementary material, which is available to authorized users.

## Background

Poly-γ-glutamic acid (γ-PGA) is a group of natural polymers consisting of l- and/or d-glutamic acid monomers polymerized through γ-glutamyl bonds [1]. Due to its features of cation chelating, hygroscopicity, water-solubility, biodegradability, and non-toxic towards humans and the environment, γ-PGA has been widely used in foods, medicine, cosmetics, and agriculture industries [2]. The bacteria *Bacillus* has been used as an efficient γ-PGA producer through various metabolically engineering manipulations [3–5]. In those studies, however, glucose and glutamate are commonly used as substrates, resulting in high production cost [1]. It is therefore necessary to use cost-effective substrates for the production of γ-PGA.

The biodiesel-derived glycerol is an ideal substrate for γ-PGA production due to its abundance and low price. About 10 kg of crude glycerol will be generated from every 100 kg of biodiesel production [6]. The rapid development of biodiesel industry has produced a large amount of crude glycerol, which makes this chemical as under-valued material [7]. There are some chemical applications utilizing pure glycerol as feedstock, but it is not economical to refine the crude glycerol into pure glycerol [8]. Crude glycerol is biologically converted into the value-added products such as acetoin [9], succinate [10], *n*-butanol [11], 3-hydroxypropionic acid [12], 1,3-propanediol [13], and poly-3-hydroxybutyrate [14]. The bioconversion of glycerol will provide an opportunity to recycle the waste disposal and produce valuable chemicals. Moreover, glycerol can generate more reducing agents than other carbon sources, and thus is able to produce high amounts of biochemical products [6].

**Fig. 1:**
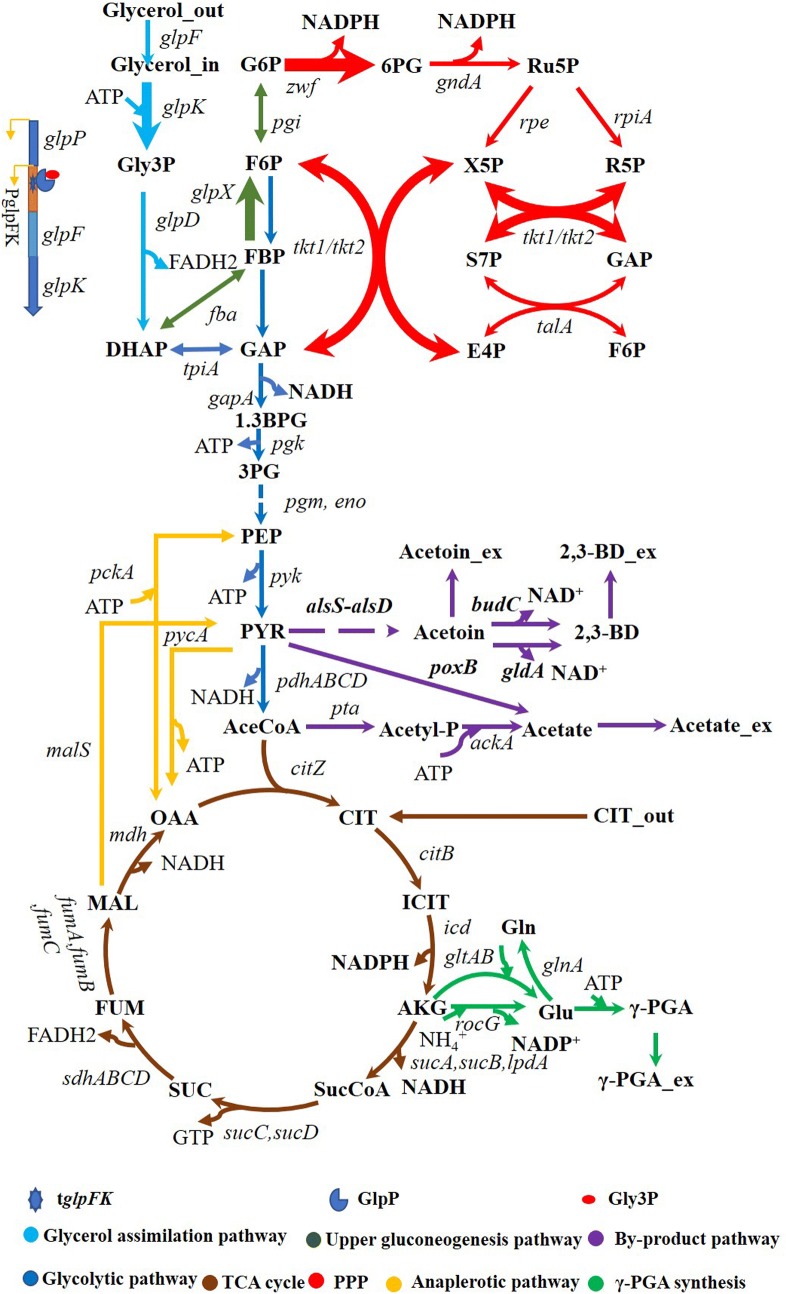
A schematic diagram of γ-PGA synthesis from glycerol in *B. licheniformis* and genetic modification strategies for producing γ-PGA employed in this study. Relevant reactions are represented by the genes. Dotted lines illustrate multiple steps, and bold lines denote over-expression of genes

Recently, using glycerol for γ-PGA production in *B. licheniformis* has been reported [15–17]. The glycerol metabolism in *B. licheniformis* WX-02 is mainly mediated by GlpK pathway [17]. Glycerol is firstly imported into the cells via glycerol transport facilitator (GlpF), and then converted to glycerol-3-phosphate (Gly3P) by glycerol kinase (GlpK), and further oxidized to dihydroxyacetone phosphate (DHAP) by Gly3P dehydrogenase (GlpD) (Fig. 1). In general, the catabolic flux of glycerol bifurcates at the DHAP node, where the gluconeogenic and glycolytic fluxes, respectively, moves towards the pentose phosphate pathway (PPP) and tricarboxylic acid (TCA) cycle (Fig. 1). Although the metabolic pathway demonstrates the possibility of using glycerol as the substrate for γ-PGA synthesis, the main drawback for this conversion pathway is the low glycerol assimilation efficiency [17]. The low activity of GlpK is proven to be the limiting factor for the glycerol metabolism in many microorganisms [18–20], and the expression level of *glpK* is induced by Gly3P and repressed by rapidly metabolizable sugars/fructose-1,6-bisphosphate (FBP) [18]. The low formation of glucose 6-phosphate (G6P) is another limiting step for glycerol metabolism. The insufficient G6P leads to scant flux towards PPP which supplies the NADPH and ribose 5-phosphate (Ru5P) for cellular biosynthetic processes [21]. Thus, the activation of GlpK and gluconeogenic pathway is a promising way to improve glycerol assimilation for γ-PGA production in *B. licheniformis.*

NADPH is proven to be an important cofactor for γ-PGA biosynthesis [22] and is mainly generated from PPP in *B. licheniformis*. When glycerol is used as the substrate, the PPP may be weakened in several ways to cause a shortage of NADPH [20]. For instance, the activities of isocitrate dehydrogenase (ICDH, EC 1.1.1.42), glucose-6-phosphate dehydrogenase (Zwf, EC 1.1.1.49), and 6-phosphogluconate dehydrogenase (Gnd, EC 1.1.1.44) are decreased compared to the glucose as the substrate [20]. The shortage of NADPH may be another reason for the insufficient production of γ-PGA from glycerol. Moreover, NADPH can prevent from cell damage resulting from reactive oxygen species [20, 23]. When cells are cultured in crude glycerol, the insufficient NADPH generation may aggravate the inhibitory effects of impurities of crude glycerol such as methanol, salts, fatty acids, and heavy metals on cell proliferation and metabolism [24]. Collectively, improvement of NADPH supply is required for γ-PGA synthesis regarding crude glycerol utilization as feedstock.

*Bacillus licheniformis* WX-02 strain has been proven as an efficient γ-PGA producer [25]. However, insufficient production of γ-PGA from glycerol was still unsolved using glycerol as a substrate [17]. The aim of this work is to improve γ-PGA production by genetically modifying the glycerol assimilation pathway, gluconeogenesis steps, and the availability of NADPH in *B. licheniformis* when using glycerol as substrate. To gain an insight into mechanism for the γ-PGA overproduction, the gene expression, intracellular metabolites, and flux distributions in the wild-type strain and the engineered strain were studied.

## Results

### Improve glycerol kinase expression to increase glycerol assimilation efficiency

Previous research demonstrated that the activity of glycerol kinase was the limiting step for glycerol assimilation in many microorganisms [18–20]. Because of the insufficient coordination of the glycerol kinase, the low glycerol assimilation efficiency limits cell growth and metabolic production [18–20]. To improve the expression of glycerol kinase, the expression levels of *glpK*, *glpP*, and *t*_*glpFK*_ were modulated. As a terminator-dependent carbon catabolite repression (CCR) mechanism is operative for the *glpFK* operon by the terminator *t*_*glpFK*_, whose formation is prevented by the Gly3P-activated antiterminator GlpP [18], the terminator *t*_*glpFK*_ was knocked out by homologous recombination, and the *glpK* and *glpP* were overexpressed, respectively, by introducing an additional copy of each gene under P43 promoter.

**Fig. 2:**
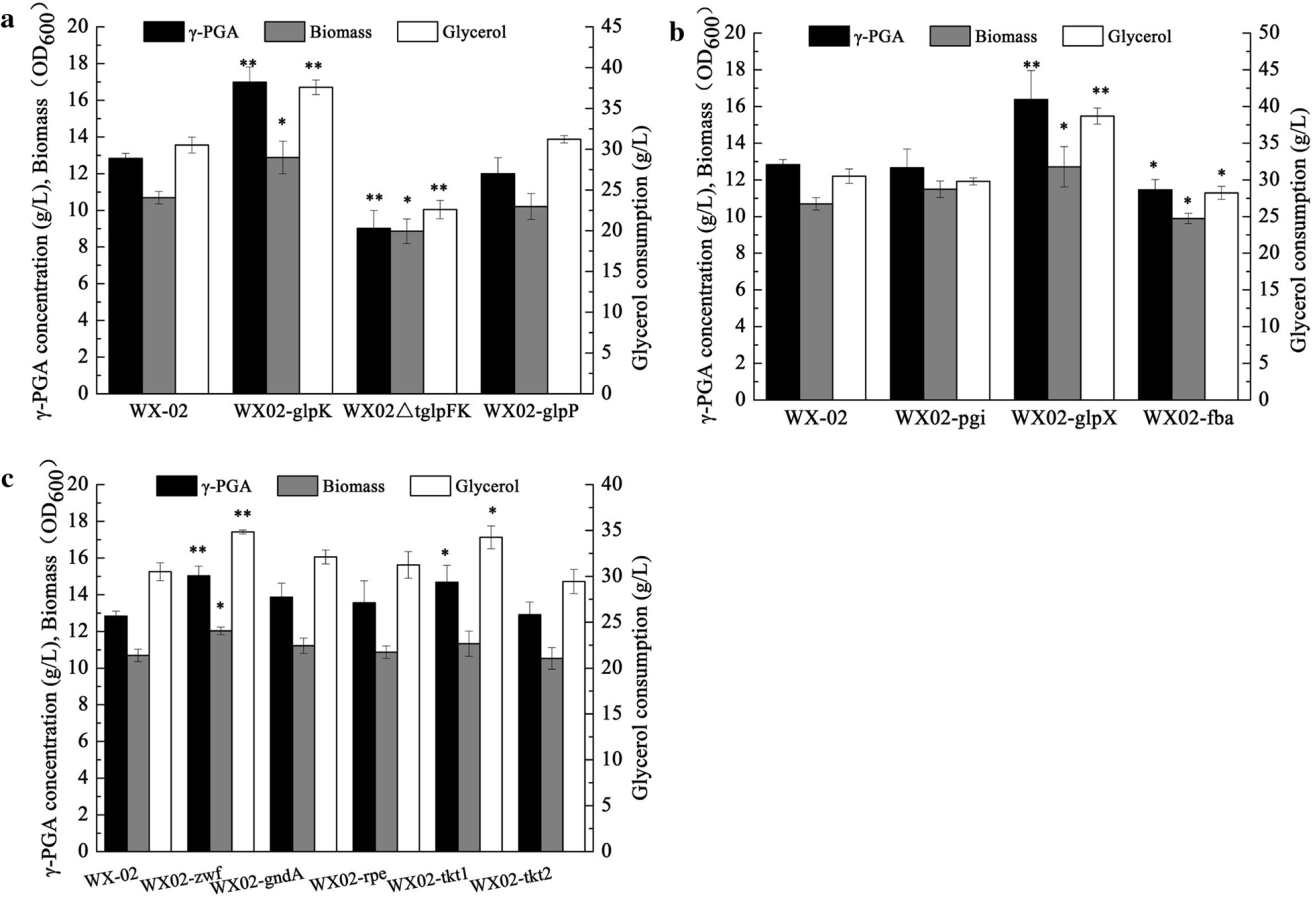
The γ-PGA production, biomass, and glycerol consumption by mutant strains using glycerol as a carbon source at 48 h. **a** Mutant strains involved in glycerol catabolism pathway; **b** mutant strains involved in gluconeogenesis pathway; **c** mutant strains involved in pentose phosphate pathway. Data are represented as the means of three replicates and bars represent the standard deviations. *P < 0.05 and **P < 0.01 indicate the significance levels between original WX-02 and its mutant strains

Based on our results, the glycerol consumption (37.59 g/L) and γ-PGA titer (16.98 g/L) of WX02-*glpK* were increased by 23.20% and 32.35%, respectively, compared to the original strain WX-02, and the growth rate of WX02-*glpK* strain increased obviously (Fig. 2a). However, removal of *t*_*glpFK*_ presented a negative effect on γ-PGA synthesis. Deletion of *t*_*glpFK*_ changed the 5′ non-coding region of *glpFK* operon, which might decrease the expression *glpF* and *glpK* and further result in the reductions of glycerol utilization and γ-PGA production. Although Gly3P-activated antiterminator GlpP could prevent the operation of the terminator *t*_*glpFK*_ [18], however, there was no noticeable influence on glycerol consumption and γ-PGA production by increasing expression of *glpP* (Fig. 2a). Based on these above results, the intracellular Gly3P might be the limiting factor for antitermination of *t*_*glpFK*_. Collectively, these results show that introducing an additional copy of *glpK* was an efficient approach to improve the glycerol assimilation and γ-PGA titer.

### Identification of the limiting enzyme in the gluconeogenesis pathway

Insufficient G6P generation in the absence of glucose leads to scant flux towards PPP. To identify limiting enzymes in upper gluconeogenesis pathway for γ-PGA synthesis, each of genes (*pgi*, *glpX* and *fba*) was overexpressed individually, and the resultant strains were denoted as WX02-*pgi*, WX02-*glpX*, and WX02-*fba*, respectively. As shown in Fig. 2b, over-expression of *glpX* significantly increased γ-PGA titer (27.67%) and glycerol consumption (26.81%) compared to the wild-type strain. However, the glycerol utilization efficiency and γ-PGA titer were decreased obviously in WX02-*fba* strain. Strengthening of *fba* could increase the amount of FBP, which further stimulated the kinase function of HprK/P and triggered the CcpA-dependent CCR mechanism [18]. As a result, the glycerol metabolism was reduced in *fba* overexpressed strain. Additionally, over-expression of *pgi* had no significant effect on γ-PGA production. Collectively, this experiment identified GlpX, the second enzyme in the upper gluconeogenesis pathway, as a bottleneck in glycerol metabolism, and this gene was considered as the metabolic engineering target for γ-PGA overproduction.

### Strengthening pentose phosphate pathway flux to enhance γ-PGA production

Strengthening PPP flux provides more NADPH supply, which may be beneficial for product biosynthesis [21]. In this study, the effects of over-expression of these five genes (*zwf*, *gnd*, *rpe*, *tkt*1, and *tkt*2) (Fig. 1) on γ-PGA synthesis were investigated. As shown in Fig. 2c, the highest γ-PGA titer of 15.03 g/L was obtained by overexpressing *zwf*, which was 17.14% enhancement over the wild type. Also, the glycerol consumption of WX02-*zwf* was increased by 14.19%. Compared to the wild type, the γ-PGA titer was increased by 14.42% in WX02-*tkt*1 strain; while over-expression of *gnd*, *tkt*2, and *rpe* presented almost no influence on the γ-PGA synthesis and glycerol consumption (Fig. 2c). Thus, the Zwf and Tkt1 were chosen to direct our subsequent metabolic engineering efforts to boost γ-PGA production.

### Combinatorial over-expression of *glpK*, *glpX*, *zwf*, and *tkt*1 genes for enhancement of γ-PGA synthesis

The combinatorial effects of the multiple genes (*glpK*, *glpX*, *zwf*, and *tkt*1) were further evaluated through multiple gene integration into the chromosome at the same time. As shown in Table 1, different combinations of the four genes gave rise to distinct results. Compared to the WX02-*glpK* (the best γ-PGA producer through single gene over-expression), slightly increase in γ-PGA titer was found in the *glpK* and *glpX* combinatorial overexpressing strain, BC2. The γ-PGA titer in the triple gene (*glpK*, *glpX*, and *zwf*) combinatorial overexpressing strain BC3 was increased by 11.01% than that of WX02-*glpK* (Table 1). The highest γ-PGA titer of 19.20 g/L was obtained by overexpressing *glpK*, *glpX*, *zwf*, and *tkt*1 concurrently, which exhibited a 49.65% enhancement over that of wild-type strain. Compared to the wild-type strain, the γ-PGA productivity increased to 0.40 g/L/h and the glycerol consumption rate and cell growth were significantly higher (Table 1). These results suggested that enhancing glycerol assimilation, gluconeogenic pathway, and the availability of NADPH led to higher titer and productivity of γ-PGA.

**Table 1:** Comparison of γ-PGA fermentation between WX-02 and mutant strains

*B. licheniformis* strains	Biomass (OD_600_)	Glycerol consumption (g/L)	γ-PGA titer (g/L)
WX-02	10.99 ± 0.34	30.51 ± 0.98	12.83 ± 0.27
BC2	12.39 ± 0.65	37.17 ± 0.85	17.42 ± 1.17
BC3	13.35 ± 0.32	38.95 ± 0.92	18.85 ± 0.53
BC4	13.33 ± 0.69	40.19 ± 0.73	19.20 ± 1.57

Strains were grown in 250-mL flasks containing 50-mL medium and incubated in a rotary shaker with 230 rpm at 37 °C for 48 h. The initial glycerol concentration was 60 g/L. Data are presented as mean ± SDs of three replicates

### Combinatorial over-expression of *glpK*, *glpX*, *zwf*, and *tkt*1 triggers metabolic flux redistribution

**Fig. 3:**
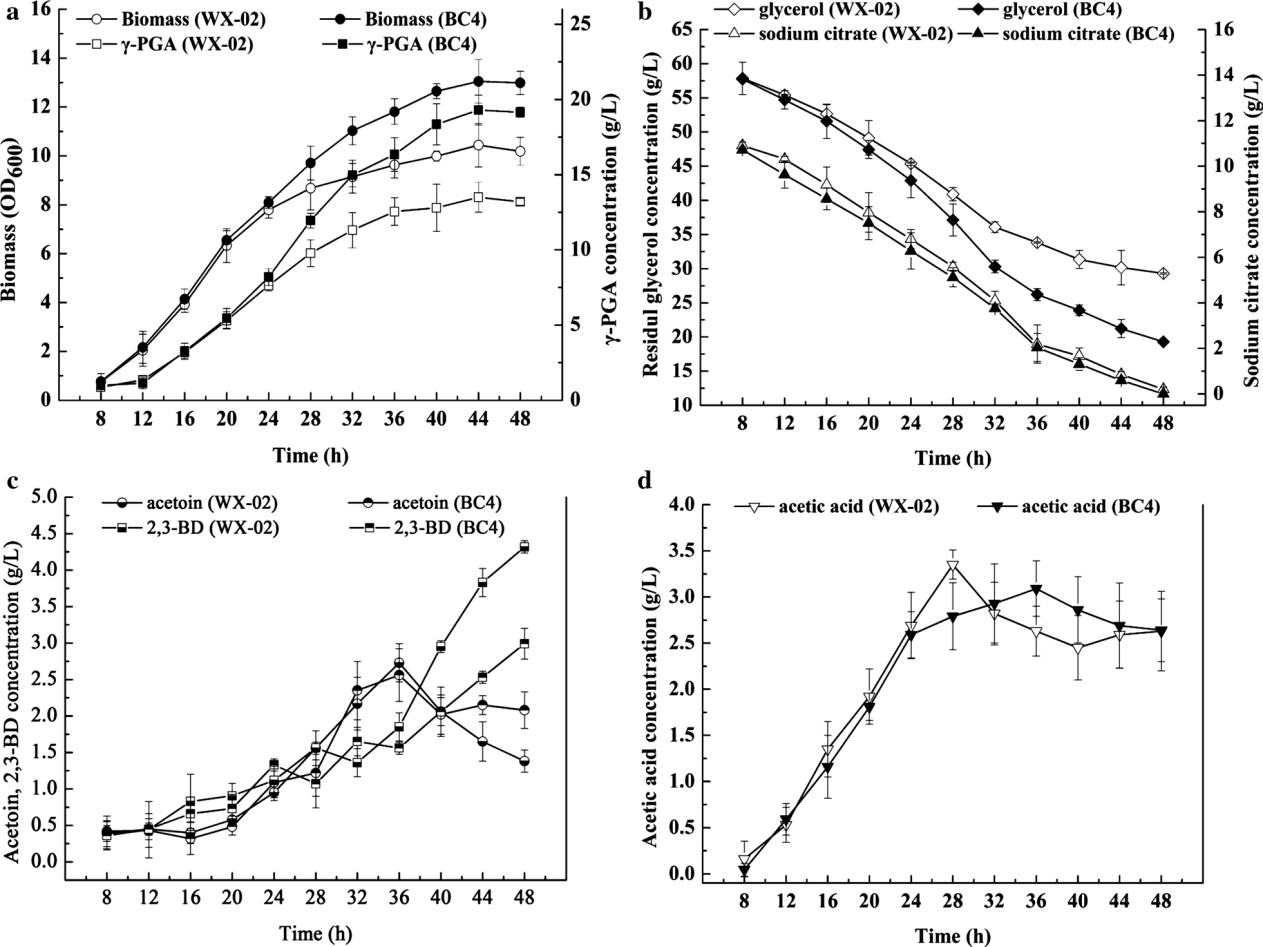
Fermentation profile of *B. licheniformis* strains BC4 and WX-02: **a** biomass density and γ-PGA titer; **b** residual glycerol and sodium citrate; **c** acetoin and 2,3-BD titer; **d** acetic acid production in shake flask containing 60 g/L crude glycerol as carbon source at 37 °C and 220 rpm. Data are represented as the means of three replicates and bars represent the standard deviations

**Fig. 4:**
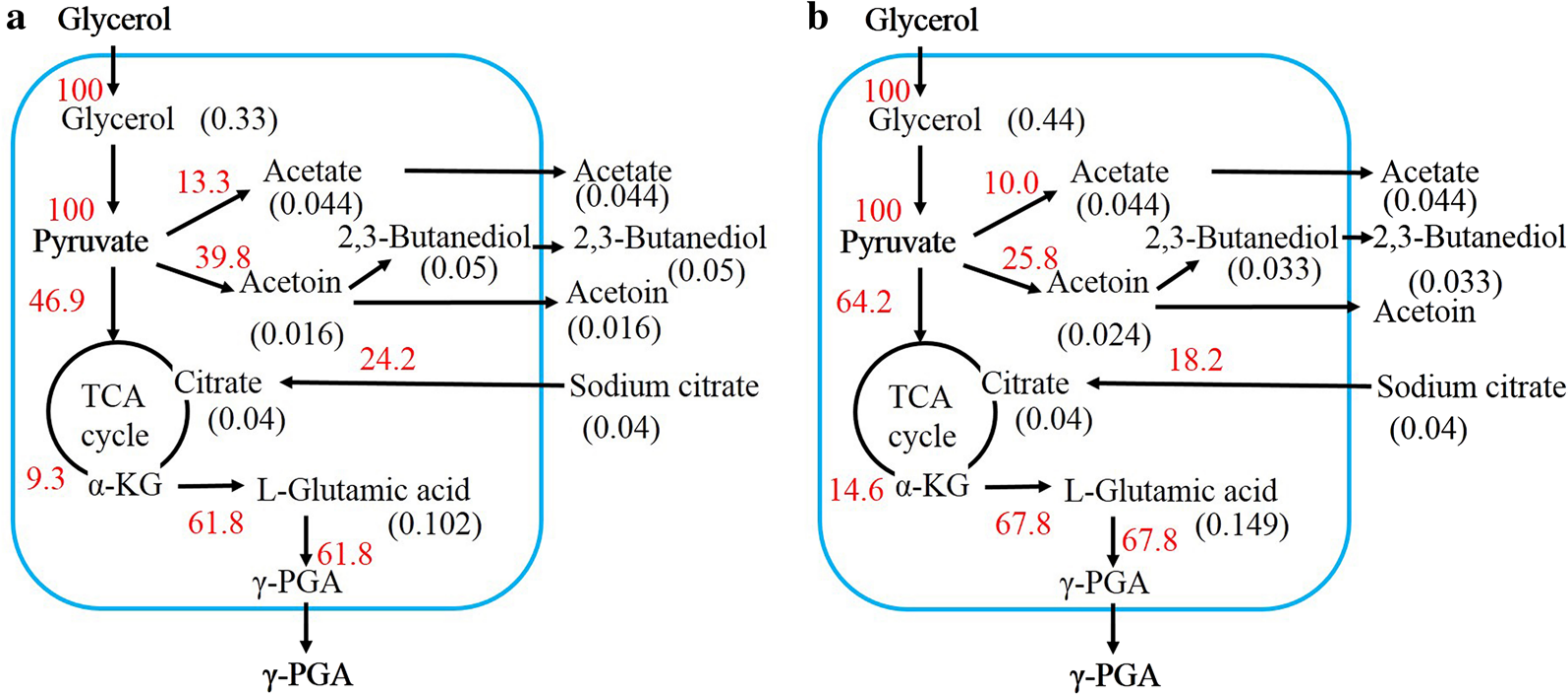
Metabolic flux redistribution of the wild-type strain (**a**), and recombinant BC4 (**b**). The concentration of γ-PGA was converted to the amount of glutamate and was calculated as followed: the amount of γ-PGA × 147/129. Data are represented as the means of three replicates and bars represent the standard deviations

To further evaluate the effects of combinatorial over-expression of quadruple genes on cellular metabolism, the fermentation characteristics of BC4 and WX-02 strains were monitored (Fig. 3). The strain BC4 utilized glycerol faster with concurrent faster cellular growth than wild-type WX-02, while citrate consumption rate was consistent with that of the wild-type strain. BC4 reached the highest cell growth at 44 h with an OD_600_ of 13.05, increasing by 25%, and the highest γ-PGA titer of the BC4 was increased by 42.96%. In addition to γ-PGA, the major metabolites of glycerol fermentation include acetoin (AC), 2,3-butanediol(2,3-BD), and acetate. Metabolic fluxes of the wild-type strain, and recombinant BC4 were calculated based on the determination of major metabolite concentrations (Fig. 4). In the WX-02 strain, the 53.1% of glycerol was converted to AC/2,3-BD (39.8%) and acetate (13.3%). The residual glycerol was directed into the TCA cycle, and the carbon flux (glycerol + citrate) towards γ-PGA was 62% (Fig. 4a). In strain BC4, however, the carbon fluxes towards AC, 2,3-BD, and acetate decreased obviously. Meanwhile, more carbon flux was channeled to TCA cycle for γ-PGA synthesis and cell growth, and the yield of γ-PGA to glycerol (0.47 g/g) was increased by 9.3% compared to wild-type strain (Fig. 4b).

Cofactors such as NADH and NADPH play important roles in regulating redox relevant reactions and generating energy. NADH is involved in ATP generation of oxidative phosphorylation and NADH-dependent products synthesis, while NADPH is essential for γ-PGA biosynthesis [26, 27]. The intracellular NADPH and NADH pools of BC4 and WX-02 strains were also compared (Additional file 1). The strain BC4 produced 20.05 μmol/gDCW NADPH, a 30.45% increase than that of wild-type strain (15.37 μmol/gDCW), while the NADH content was decreased by 20%. The ratio of NADPH/NADH was 2.05, increased by 62.70%. These results suggested that combinatorial over-expression of quadruple genes might affect the overall metabolic network, and further led to the metabolic flux redistribution.

### Transcriptional levels and intracellular metabolites analysis

**Fig. 5:**
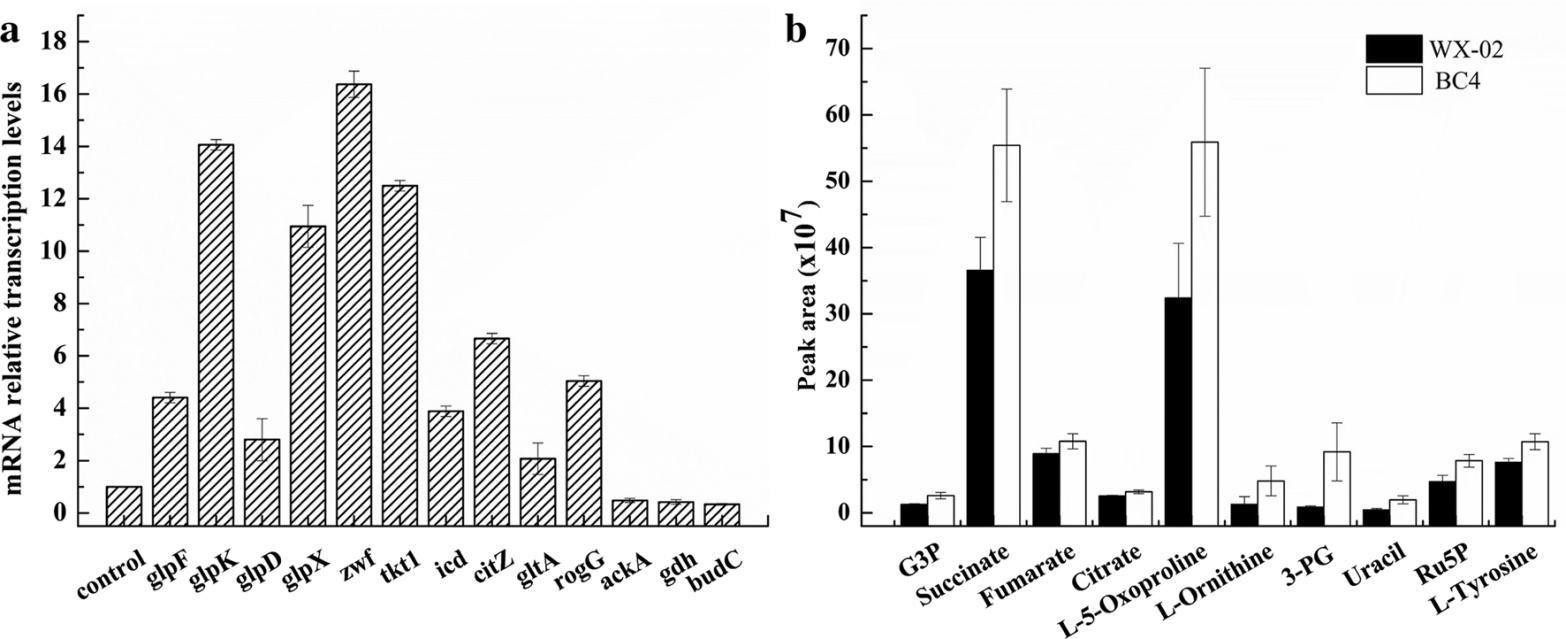
The transcriptional levels of selected genes in BC4 and WX-02 strains (**a**), and comparison of intracellular metabolites of BC4 and WX-02 strains (**b**). Data are represented as the means of three replicates and bars represent the standard deviations

To investigate the transcriptional responses to γ-PGA overproduction, the transcription levels of related genes of the BC4 and WX-02 were measured by qRT-PCR (Fig. 5a). Compared with WX-02, the transcriptional levels of *glpK*, *glpX*, *zwf*, and *tkt*1 in BC4 strain were up-regulated by 13.06-, 9.95-, 15.37-, and 11.49-folds, respectively. The four enzyme activities were measured using the crude extract. We found that glycerol kinase, glucose-6-phosphate dehydrogenase, FBPase, and transketolase activities were increased by 18.62%, 23.23%, 65.99%, and 68.98% (*P* < 0.05), compared to those in wild-type strain, respectively (Additional file 2). The expression levels of *glpF* and *glpD* of BC4 strain were 4.4- and 2.8-folds of those of wild type, respectively, which might provide the explanation for higher glycerol consumption in strain BC4. The two genes *icd* and *citZ* involved in TCA cycle (Fig. 1) were up-regulated by 2.8-fold and 5.66-fold than those of wild type. The α-ketoglutaric acid could be converted to glutamate by glutamate dehydrogenase encoded by *rocG* [22]. Interestingly, the expression levels of *rocG* and *gltA* in BC4 were increased by 4.04-fold and 1.07-fold, respectively. Meanwhile, the expression levels of *ackA*, *alsS*, *alsD*, *gldA*, and *budC* genes responsible for acetate, AC, and 2,3-BD biosynthesis were all decreased.

To gain a further insight into mechanisms for the γ-PGA overproduction, the intracellular metabolite concentrations in WX-02 and BC4 were measured by GC–MS-based metabolome approach [28, 29]. Based on our results, the metabolites (containing intermediates of glycolysis, PPP, TCA cycle, and amino acids pools) with significant differences among the two strains were listed in Fig. 5b. In terms of the glycerol pathway, the pool sizes of Gly3P in engineered strains were higher than that of the WX-02. The metabolites involved in the PPP, such as Ru5P, uracil, and tyrosine, were increased in engineered strains (increased by 40.60–328.66%). TCA cycle metabolites, such as succinate, fumarate, and citrate, showed a higher abundance in the mutants compared to the wild-type strain WX-02. The glycolysis metabolite 3-phosphoglyceric acid (3PG) was increased by 9.35-fold. These results were consistent with the above-performed transcription analysis, explaining γ-PGA production enhancement. Due to the difficulty in analyzing glycolytic intermediates, some metabolites have not been detected like G6P, F6P, and FBP in cell extracts.

### γ-PGA production by recombinant BC4 strain with crude glycerol as substrate

The ultimate goal of this work is to produce γ-PGA using crude glycerol and provides a solution for high cost of γ-PGA production. The alkaline crude glycerol (CG_base_ 78 g/L) and acid crude glycerol (CG_acid_ 90 g/L) were used to analyze the influence of crude glycerol on γ-PGA production by the recombinant strain BC4. Equivalent pure glycerol (60 g/L) was used as the control group. When CG_base_ was used as the carbon source, strain BC4 showed a high productivity of 0.38 g/L/h, and produced 18.41 g/L γ-PGA, with a high yield of 0.46 g γ-PGA/g glycerol (Table 2). However, the BC4 strain produced 16.50 g/L γ-PGA and a correspondent productivity of 0.34 g/L/h when the CG_acid_ was served as the carbon source (Table 2).

**Table 2:** Comparison of γ-PGA production by *Bacillus* strains using glycerol as substrate

Strains	Main nutrients (g/L)	Culture time (h)	γ-PGA titer (g/L)	γ-PGA productivity (g/L/h)	References
*B. licheniformis* ATCC 9945a	l-Glutamic acid, glycerol, citric acid, NH_4_Cl	96	23	0.24	[15]
*B. licheniformis* CCRC 12826	As above	96	19.62	0.2	[16]
*B. licheniformis* WBL-3	As above	88	22.8	0.26	[37]
*B. subtilis* RKY3	l-Glutamic acid, glycerol, citric acid, NH_4_Cl, yeast extract	24	48.7	2.03	[33]
	l-Glutamic acid, glycerol, citric acid, NH_4_Cl	48	28.4	0.59	[35]
*B. subtilis* IFO 3335	l-Glutamic acid, glycerol, citric acid, (NH_4_)_2_SO_4_	96	10–20	0.1–0.21	[45]
*B. subtilis* BL53	l-Glutamic acid, glycerol, citric acid, NH_4_Cl	72	10.4	0.14	[46]
	As above	96	17.0	0.18	[47]
*B. licheniformis* WX02-P43*glpFK*	Sodium glutamate, crude glycerol, citric acid, NH_4_Cl	48	16.63	0.35	[17]
*B. methylotrophicus* SK19.001	Glycerol, sodium citrate, peptone	66	35.3	0.535	[36]
*B. subtilis* C1	Glycerol, citric acid, NH_4_Cl	144	21.4	0.15	[38]
*B. licheniformis* WX-02	Glycerol, sodium citrate, NaNO_3_, NH_4_Cl	48	12.83	0.27	This study
*B. licheniformis* BC4	As above	48	19.20	0.4	This study
*B. licheniformis* BC4	CG_base_, sodium citrate, NaNO_3_, NH_4_Cl	48	18.41	0.38	This study
*B. licheniformis* BC4	CG_acid_, sodium citrate, NaNO_3_, NH_4_Cl	48	16.5	0.34	This study

CG_acid_ means acid crude glycerol; CG_base_ means alkaline crude glycerol

## Discussion

Glycerol has been considered as a promising feedstock for biochemicals production due to its abundance, low price, and high degree of reduction [6]. Much efforts have been made to engineer glycerol catabolism and downstream metabolic pathways for production of various valuable products in *E. coli*, *Corynebacterium glutamicum*, *Klebsiella* species, and *Clostridium species* [8, 13, 30–32]. Nevertheless, few related studies have been performed in *B. licheniformis* [17, 33]. As a consequence, the metabolic pathway of *B. licheniformis* was rationally engineered for efficient production of γ-PGA from crude glycerol (Fig. 1).

In this study, the gene *glpK* in *B. licheniformis* was overexpressed by using three different methods to increase glycerol assimilation efficiency. According to our results, introducing an additional copy of *glpK* was an efficient approach to improve glycerol consumption and γ-PGA yield. With one added copy of *glpK* on the chromosome, the *glpK* transcription level and GlpK enzyme activity were increased by 12.06-fold and 19%, respectively, compared to the wild-type strain. The expression levels of *glpF* and *glpD* genes were also found to be up-regulated in *glpK* overexpressing strain due to an increase in Gly3P generated by GlpK (Fig. 5b). These results were consistent with the previous reports [19, 20], and indicated that the GlpK was a rate-limiting step in glycerol metabolism in *B. licheniformis.* Another limiting step in glycerol metabolism is the necessity for formation of G6P through gluconeogenic pathway. In this study, the limiting enzymes in upper gluconeogenesis pathway were identified in *B. licheniformis* cultured with glycerol. Over-expression of *glpX* obviously improved glycerol assimilation efficiency and γ-PGA accumulation, which might be due to the fact that the increasing G6P pool was beneficial for enhancing the PPP flux.

The supply of NADPH, which has been proven to be critical for γ-PGA synthesis, may be another limited factor. Several attempts have been conducted to increase the availability of NADPH by overexpressing glucose 6-phosphate dehydrogenase (encoded by *zwf*) and 6-phosphogluconate dehydrogenase (encoded by *gnd*) [4, 21]. The roles of Zwf and Gnd in γ-PGA synthesis from glycerol were evaluated in this study. Our results implied that over-expression of *zwf* had the positive effect on production. Similarly, over-expression of *zwf* led to a 35% improvement of γ-PGA titer using glucose as carbon source [4]. Apart from the Zwf and Gnd, the ribulose-5-phosphate 3-epimerase and transketolase were also studied. Although two transketolase isoenzymes are preserved in *B. licheniformis*, the transketolase encoded by *tkt*1 was firstly identified as the dominant isoenzyme and overexpressed for improving γ-PGA biosynthesis in *B. licheniformis.* Also, over-expression of transketolase in *E. coli* strain could improve the phenylalanine production from glycerol [19]. However, over-expression of *rpe* had almost no influence on the γ-PGA production and glycerol consumption. These results indicated that more precursors and NADPH might be generated after over-expression of *zwf* and *tkt*1.

In this study, the combinatorial effects of over-expression of *glpK*, *glpX*, *zwf*, and *tkt*1 on γ-PGA titer were evaluated. In the presence of pure glycerol, the recombinant strain BC4 displayed a 49.65% increase in γ-PGA titer and 31.76% increase in glycerol consumption, compared to the wild-type strain. The circuit of the γ-PGA overproduction in the BC4 strain was further investigated. In the present study, the transcriptional levels of genes involved in glycerol catabolic pathway were enhanced in BC4 strain, as well as the GlpK activity and Gly3P accumulation (Fig. 5b), and these results were consistent with the improvement of glycerol consumption. Additionally, TCA cycle is an essential part of γ-PGA synthetic pathways and cell growth [34]. In BC4 strain, more carbon flux was channeled to TCA cycle and glutamate for γ-PGA biosynthesis (Figs. 4 and 5b). The transcription of genes and concentrations of metabolites involved in TCA cycle and glutamate biosynthesis were increased obviously (Fig. 5a, b), further demonstrating that more carbon flux was channeled towards TCA cycle to generate sufficient ATP and glutamate for γ-PGA synthesis.

In NADH-dependent production systems, NADH availability and the ratio of NADH/NAD^+^ may play a pivotal role in metabolic regulation [9]. Over-expression of *glpK*, *glpX*, *zwf*, and *tkt*1 was accompanied with the cofactor conversion from NADH to NADPH. The decreasing of NADH might result in reduction of 2,3-BD synthesis. The decreased overflow metabolism may help reducing the carbon waste and boosting carbon flux towards γ-PGA. Moreover, over-expression of *glpX*, *zwf*, and *tkt*1 caused an obvious flux redirection at the G6P node, and enhancement of PPP flux in comparison to the wild-type strain (Fig. 5a, b). These results suggested that combinatorial over-expression of quadruple genes could change the metabolic network, and redistribute metabolic flux to γ-PGA production.

The use of crude glycerol for microbial γ-PGA production not only reduces the cost of γ-PGA production, but also provides a solution for the excess crude glycerol from biodiesel production. This study successfully developed a recombinant strain BC4 for efficient production of γ-PGA from crude glycerol. As shown in Table 2, the final γ-PGA yield was 0.46 g/g glycerol in the glutamic acid-free medium containing crude glycerol, sodium citrate, NaNO_3_, NH_4_Cl, and the γ-PGA yield reached 68.2% of theoretical yield, which exhibits a significant increase compared to the wild-type strain. The γ-PGA titer (18.41 g/L) produced by BC4 was slightly lower than those produced by many γ-PGA producers [15, 16, 33, 35–37]; however, the cost of feedstocks was the lowest value compared to other strains. Moreover, compared with the pure glycerol, by using the industry byproduct as feedstock, nearly a half of material cost could be saved (Additional file 3). To the best of our knowledge, the productivity of γ-PGA achieved in this study is the highest value reported using crude glycerol as the substrate [17, 38]. Our results implied that the crude glycerol might be an ideal substitute for glucose for commercial production of γ-PGA using the metabolically engineered *B. licheniformis*.

## Conclusions

The metabolic pathway of *B. licheniformis* was rewired to improve γ-PGA production from crude glycerol. After systematically investigating the three module pathways genes *glpP*, *t*_*glpFK*_, *glpK*, *fba*, *glpX*, *pgi*, *zwf*, *gnd*, *rpe*, *tkt*1, and *tkt*2, we confirmed that GlpK, GlpX, Zwf, and Tkt1 were the key enzymes for γ-PGA synthesis in glycerol fermentation. Through combinational expression of these key genes, the titer and productivity of γ-PGA was increased to 19.20 g/L and 0.4 g/L/h, respectively, which were about 1.50-fold of those of the wild-type strain. We also achieved the high γ-PGA titer of 18.4 g/L using crude glycerol as substrate, comparing with other studies reported, with the highest γ-PGA productivity of 0.38 g/L/h. Our studies successfully showed that the crude glycerol can be efficiently converted to γ-PGA by metabolically engineered *B. licheniformis*, and the developed strategy might be applied to high-level production of other valuable compounds from crude glycerol.

## Methods

### Bacterial strains, media, and culture conditions

Bacterial strains and plasmids used in this study are presented in Table 3, the primers listed in Additional file 4 were designed on the basis of *B. licheniformis* WX-02 genome sequence [GenBank: AHIF00000000] [39]. *E. coli* DH5α and *B. licheniformis* WX-02 (CCTCC M208065) were used for plasmid construction and γ-PGA production, respectively. *E. coli* DH5α and *B. licheniformis* strains were grown in Luria–Bertani (LB) medium for genetic manipulation and seed culture. When necessary, the medium was added with appropriate kanamycin (20 μg/L).

**Table 3:** Strains and plasmids in this study

Strains	Characteristic	Source
*B. subtilis* 168	Wild type	ATCC
*B. licheniformis*		
WX-02	Wild-type strain, CCTCC M208065	CCTCC
WX02-*glpK*	WX-02 derivative, over-expression of *glpK*	This study
WX02-*glpP*	WX-02 derivative, over-expression of *glpP*	This study
WX02∆t_*glpFK*_	WX-02 derivative, defective in *t*_*glpFK*_	This study
WX02-*zwf*	WX-02 derivative, over-expression of *zwf*	This study
WX02-*tkt*1	WX-02 derivative, over-expression of *tkt*1	This study
WX02-*tkt*2	WX-02 derivative, over-expression of *tkt*2	This study
WX02-*rpe*	WX-02 derivative, over-expression of *rpe*	This study
WX02-*gndA*	WX-02 derivative, over-expression of *gndA*	This study
WX02-*pgi*	WX-02 derivative, over-expression of *pgi*	This study
WX02-*fba*	WX-02 derivative, over-expression of *fba*	This study
WX02-*glpX*	WX-02 derivative, over-expression of *glpX*	This study
BC2	WX02-*glpK* derivative, over-expression of *glpX*	This study
BC3	BC2 derivative, over-expression of *zwf*	This study
BC4	BC3 derivative, over-expression of *tkt*1	This study
*E. coli* DH5a	F^−^Φ80d/*lacZ*ΔM15, Δ(*lacZYA*-*argF*)U169, *recA*1, *endA*1, *hsdR*17 (r_K_^−^, m_K_^+^), *phoA*, *supE*44, λ^−^, *thi*-1, *gyrA*96, *relA*1	Laboratory stock
Plasmids		
T_2_(2)-ori	*Bacillus knockout vector; Kan* ^*r*^	Laboratory stock
T_2_-G*glpK*	T_2_(ori)-*glpK*(A+B+*glpK*); to over-express *glpK*	This study
T_2_-G*glpP*	T_2_(ori)-*glpP*(A+B+*glpP*); to over-express *glpP*	This study
T_2_-∆*t*_*glpFK*_	T_2_(ori)-*t*_*glpFK*_(A+B); to knock out *t*_*glpFK*_	This study
T_2_-G*zwf*	T_2_(ori)-*zwf*(A+B+*glpK*); to over-express *glpK*	This study
T_2_-G*tkt*1	T_2_(ori)-*tkt*1(A+B+*tkt*1); to over-express *tkt*1	This study
T_2_-G*tkt*2	T_2_(ori)-*tkt*2 (A+B+*tkt*2); to over-express *tkt*2	This study
T_2_-G*rpe*	T_2_(ori)-*rpe*(A+B+*rpe*); to over-express *rpe*	This study
T_2_-G*gndA*	T_2_(ori)-*gndA*(A+B+*gndA*); to over-express *gndA*	This study
T_2_-G*pgi*	T_2_(ori)-*pgi*(A+B+*pgi*); to over-express *pgi*	This study
T_2_-G*glpX*	T_2_(ori)-*glpX*(A+B+*glpX*); to over-express *glpX*	This study
T_2_-G*fba*	T_2_(ori)-*fba*(A+B+*fba*); to over-express *fba*	This study

The fermentation for γ-PGA production was carried out as described previously [25]. The medium for γ-PGA production contained (per liter) 60 g glycerol, 12 g sodium citrate, 15 g NaNO_3_, 8 g ammonium chloride, 1 g K_2_HPO_4_·3H_2_O, 1 g MgSO_4_·7H_2_O, 1 g ZnSO_4_·7H_2_O, 1 g CaCl_2_, and 0.15 g MnSO_4_·H_2_O with pH 7.2. *B. licheniformis* recombinants were cultivated in 250 mL Erlenmeyer’s flasks containing 50 mL medium at 37 °C and 230 rpm for 48 h. The alkaline crude glycerol (CG_base_) with 82% purity (w/w) or acid crude glycerol (CG_acid_) with 72% purity (w/w) was added to reach 60 g/L glycerol concentration. Crude glycerol purification by treatment with activated carbon was performed according to the previous report [17].

### Gene knockout in *B. licheniformis*

The gene *t*_*glpFK*_ was deleted by homologous recombination based on the previous described method [40]. Briefly, the homologous arms of *t*_*glpFK*_ were amplified by the corresponding primers and fused by splicing overlap extension PCR (SOE-PCR). The fused fragments were then inserted into T_2_(2)-ori at the restriction sites *Xba*I/*Sac*I, generating the recombinant plasmid T_2_-∆*t*_*glpFK*_. Furthermore, the T_2_-∆*t*_*glpFK*_ was transformed into *B. licheniformis* WX-02 strain via electroporation. The positive transformants were incubated in LB medium containing kanamycin at 45 °C, and then streaked onto LB agar containing kanamycin incubation to obtain the single-crossover recombinants. The selected colonies were grown in LB medium at 37 °C with serial subcultures to obtain double crossover colonies. The gene-deleted strains were further verified by PCR and DNA-sequencing.

### Gene integration in *B. licheniformis*

The prophage regions were chosen for the integrated expression of genes in *B. licheniformis* [41]. Briefly, the homologous arms of *xkdG*, *glpX*, and *amyL* terminator were, respectively, amplified from WX-02 genome, and P43 promoter was amplified from *B. subtilis* 168 genome. The homologous arms of *xkdG*, P43 promoter, *glpX*, and *amyL* terminator were fused by SOE-PCR, then inserted into T_2_(2)-ori, resulting in the plasmid T_2_-GglpX. The integration mutants were obtained according to the gene knockout method described in above section (Additional file 5). Other gene integrated expression strains were constructed using the same method.

### Transcriptional level analysis

The total RNA was extracted by according to the previous report [25]. The DNA was digested by DNase I enzyme (TaKaRa, Japan), and RevertAid First Strand cDNA Synthesis Kit (Thermo Fisher, USA) was used for first-stand cDNA synthesis following the manufacturer’s instructions. The real-time PCR was performed by using SYBR^®^ Select Master Mix (ABI, USA). The primers listed in Additional file 4 were used for amplifying the corresponding genes, and the 16s rRNA gene was served as the internal reference gene and real-time-PCR was conducted in triplicate for each sample. The expression levels for the recombinant strains were compared to the wild-type strain after normalization.

### Determination of enzymatic activities

Cells were harvested in mid-log-phase (cultivated for 20 h), washed with cold potassium phosphate buffer, and then suspended in the same buffer. Cell disruption was achieved by sonication (150 W, 20 kHz, pulse: 2 s on; 2 s off; total: 8 min;), and disruption solution was centrifuged at 12,000×*g* for 20 min at 4 °C to remove cell debris. The supernatant was stored on 4 °C for subsequent experiments.

Activities of Zwf in crude cell extracts were measured by determining the increase in A340 of NADPH at 30 °C as described before [4]. Glycerol kinase activity was measured using a pyruvate kinase and lactate dehydrogenase coupled assay as described previously [19]. The transketolase activity was measured according to the previous described method [42]. The activity of FBPase was measured following the increase in phosphate concentration liberated using a malachite green/ammonium molybdate solution [19].

### Intracellular metabolites analysis

The cells were collected and analyzed as described previously with minor modifications [43, 44]. Briefly, an appropriate volume of culture broth was harvested at 20 h, and mixed with an equivalent volume of 0.25 M perchloric acid by vortexing transitorily. The volume of transferred cells was adjusted based on OD_600_ at each sample to satisfy the formula: sampling volume (mL) × OD_600_ = 10.0 and washed 2 times with 1.0 mL of cold 0.80% NaCl solution. 1.0 mL of pre-cooling 75% ethanol was used for cell metabolites extraction. The samples were treated at 90 °C for 10 min and subsequent at − 40 °C for 5 min, and centrifuged at 4 °C, 12,000×*g* for 5 min. Supernatants were collected and dried in a rotary vacuum centrifuge device (LABCONCO) for GC–MS analysis. In each experiment, sampling was carried out sevenfold in parallel.

Samples were derivatized before GC–MS analyzing [43]. Firstly, the extracts were oximated with 50 μL of 20 mg/mL methoxyamine hydrochloride (Sigma-Aldrich) in pyridine at 37 °C for 90 min. Before derivatization, 10 μL of 0.5% phenylethyl acetate was added as an analytical internal standard. Subsequently, the acidic protons in samples were derivatized by 37 °C reaction for 30 min with addition of 50 μL of *N*-methyl-*N*-(trimethylsilyl)-trifluoroacetamide (MSTFA, Sigma-Aldrich). 1 μL aliquot of derivatized samples was injected into a DB-5MS column (250 μm ID × 30 m, 0.25 μm, Thermo Fisher Scientific) using splitless model. The temperature-programmed profile was set as follows: 50 °C for initial temperature, increase with 10 °C/min to 110 °C and keep for 5 min, then ramp to 165 °C at a rate of 2 °C/min, lastly raise to 220 °C at a rate of 3 °C/min, and hold at 220 °C for 10 min. Helium was used as the carrier gas and the speed of the flow was kept at 1.2 mL/min constantly. For the MS aspect, electron impact ionization (EI) was selected and the ionization was of 70 eV energy with 8000 V acceleration voltage. The MS transfer line temperature and ion source temperature were 280 °C and 300 °C, respectively. The scanned range was 50–650 *m*/*z*.

The compounds were identified by comparing mass spectra to National Institute of Standards and Technology (NIST) MS search [version 2.2 (2014)]. TranceFinder 4.1 (Thermo Fisher Scientific) was used for automatic peak picking and calculation of the peak area. The selected peaks were also confirmed manually, and the peak area was normalized by internal standard. The metabolite levels were further compared based on their peak areas of GC–MS chromatogram [43]. Principal component analysis (PCA) and orthogonal partial least squares discrimination analysis (OPLS-DA) were performed by SIMCA 13 (UMETRICS, Sweden) with the metabolome data set (Additional file 6).

### Quantification of NADPH and NADH

The intracellular concentrations of NADPH and NADH were determined in the exponential growth phase according to the previous method [4]. For NADH, the reaction mixture (200 μL) contained 50 mM HEPES (pH 7.5), 2 mM EDTA, 120 μM DCPIP, 1 mM PMS, 10 μL alcohol dehydrogenase, and 20 μL disrupted cell solution. Ethanol (15 μL) was then added to the wells to start the reaction. The decrease of A600 was monitored for 5 min. The contents were calculated by reference to the standards run concurrently (0–40 pmol NADH). The same reaction mixture was applied to determine NADPH concentration; however, the reaction was started by the addition of 10 μL glucose-6-phosphate dehydrogenase. The decrease in A600 was monitored for 5 min. The rates were calculated with the standards, similar to the NADH analysis.

### Analytical procedures

Cell growth was measured at 600 nm with a spectrophotometer (Bio-Rad, USA). A standard curve relating OD to cell dry weight (CDW) was developed (1 OD_600_ = 0.363 g CDW/L). The concentrations of γ-PGA were determined using a gel permeation chromatography (GPC) system equipped with TSK Gel G6000 PWXL column (7.8 mm × 300 mm, Tosoh, Tokyo, Japan) [25]. The mobile phase was a mixture of 25 mM sodium sulfate solution: acetonitrile (8:1) at a flow rate of 0.5 mL/min and detected at 220 nm. The calibration curve for the determination of the γ-PGA concentration was established using purified γ-PGA. Samples were appropriately diluted with ethanol, and then mixed by vortex. After centrifugation, the supernatant was used for subsequent analysis. Citric acid content was determined by a high-performance liquid chromatography (HPLC) using Zorbax SB-Aq (4.6 mm ID × 250 mm, 5 μm) column by means of a UV light detector. A mobile phase of 99% 20 mM Na_2_HPO_4_ and 1% acetonitrile (pH 2.0) at a flow rate of 0.5 mL/min was used. The acetoin, 2,3-butanediol (2,3-BD), and acetate concentrations were determined by gas chromatography method as described previously [40]. Glycerol was quantified by HPLC (Agilent, USA) using Inertsil NH_2_ column (250 mm × 4.6 mm, 5 μm; GL Science Tokyo, Japan) and an evaporative light-scattering detector (ELSD, Agilent, USA). A mobile phase of 80% acetonitrile at 1.0 mL/min flow rate was used [17].

### Statistical analysis

All experiments were performed in three replicates. The data were represented as the mean value ± standard deviation. All data were conducted to analyze the variance at *P* < 0.05 and *P* < 0.01, and a *t* test was applied to compare the mean values using the Statistica 6.0 software package.

## Abbreviations

### Metabolites

Gly3P: glycerol-3-phosphate; DHAP: dihydroxyacetone phosphate; GAP: glyceraldehyde 3-phosphate; FBP: fructose 1.6-bisphosphate; F6P: fructose 6-phosphate; G6P: glucose-6-phosphate; 6PG: 6-phosphogluconate; R5P: ribulose 5-phosphate; Ru5P: ribose 5-phosphate; X5P: xylulose 5-phosphate; S7P: sedoheptulose 7-phosphate; E4P: erythrose 4-phosphate; 1,3 BPG: 1,3-bisphosphoglyceric acid; 3PG: 3-phosphoglyceric acid; PEP: phosphoenolpyruvate; PYR: pyruvate; AceCoA: acetyl-CoA; CIT: citrate; ICIT: isocitrate; AKG: 2-oxoglutarate; SUC-CoA: succinyl-CoA; SUC: succinate; FUM: fumarate; MAL: malate; OAA: oxaloacetate; Glu: glutamate; γ-PGA: poly-γ-glutamic acid; AC: acetoin; 2,3-BD: 2,3-butanediol.

### Enzymes and others

GlpP: glycerol uptake operon antiterminator; GlpF: glycerol transport facilitator; GlpK: glycerol kinase; GlpD: glycerol-3-phosphate dehydrogenase; Fba: fructose-bisphosphate aldolase; GlpX: fructose-1,6-bisphosphatase; Pgi: glucose-6-phosphate isomerase; Zwf: glucose-6-phosphate dehydrogenase; GndA: 6-phosphogluconate dehydrogenase; Rpe: d-ribulose-5-phosphate 3-epimerase; Tkt: transketolase; PYC: pyruvate carboxylase; CitZ: citrate synthase; CitB: aconitate hydratase; ICDH: isocitrate dehydrogenase; OGDC: α-oxoglutarate dehydrogenase; SucCD: succinyl-CoA ligase; SDH: succinate dehydrogenase; FH: fumarate hydratase; Mdh: malate dehydrogenase; GDH: glutamate dehydrogenase; GOGAT: glutamate synthase; PTA: phosphate acetyltransferase; AK: acetate kinase; AlsS: acetolactate synthase; AlsD: alpha-acetolactate decarboxylase; 2,3-BDH: 2,3-butanediol dehydrogenase; GDH: glycerol dehydrogenase; PPP: pentose phosphate pathway; TCA: tricarboxylic acid; CG_acid_: acid crude glycerol; CG_base_: alkaline crude glycerol; CCR: carbon catabolite repression; MSTFA: *N*-methyl-*N*-(trimethylsilyl)-trifluoroacetamide; PCA: principal component analysis; OPLS-DA: orthogonal partial least squares discrimination analysis; CDW: cell dry weight; GPC: gel permeation chromatography.

## Additional files

**Additional file 1: Table S1.** The NADPH and NADH concentrations in WX-02 and BC4.

**Additional file 2: Table S2.** Enzyme activities of GlpK, GlpX, Zwf and TKT in WX-02 and BC4.

**Additional file 3: Table S3.** Comparison of cost with pure glycerol or crude glycerol as fermentation feedstock.

**Additional file 4: Table S4.** Primers used for PCR and qRT-PCR in this study.

**Additional file 5: Figure S1.** The schematic diagram of recombination strain WX02-*glpX* construction.

**Additional file 6: Figure S2.** The result of PCA and OPLS-DA with metabolome data of the WX-02 and BC4 strains cultivated in the glycerol medium.
