# Supplementary material for: Rewiring glycerol metabolism for enhanced production of poly-γ-glutamic acid in Bacillus licheniformis
Source: Biotechnol Biofuels. 2018 Nov 9;11:306. doi: 10.1186/s13068-018-1311-9 (PMC6225680; doi:10.1186/s13068-018-1311-9)
Supplement: Supplementary file 1 — Additional file 1: Table S1. The NADPH and NADH concentrations in WX-02 and BC4. [file 13068_2018_1311_MOESM1_ESM.docx]

**Table S1 NADPH and NADH concentrations in WX-02 and BC4**

| **Strains** | **NADPH concentration**  **(μmol/g/DCW)** | **NADH concentration**  **(μmol/g/DCW)** | **NADPH/NADH** |
| --- | --- | --- | --- |
| WX-02 | 15.37±0.68 | 12.25±1.68 | 1.26±0.12 |
| BC4 | 20.05±0.39 | 9.79±0.80 | 2.05±0.13 |

The intracellular concentrations of NADPH and NADH were determined in the exponential growth phase. Data are presented as mean ± SDs of three replicates.
