# Supplementary material for: Rewiring glycerol metabolism for enhanced production of poly-γ-glutamic acid in Bacillus licheniformis
Source: Biotechnol Biofuels. 2018 Nov 9;11:306. doi: 10.1186/s13068-018-1311-9 (PMC6225680; doi:10.1186/s13068-018-1311-9)
Supplement: Supplementary file 2 — Additional file 2: Table S2. Enzyme activities of GlpK, GlpX, Zwf and TKT in WX-02 and BC4. [file 13068_2018_1311_MOESM2_ESM.docx]

**Table S2 Enzyme activities of GlpK, GlpX, Zwf and TKT in WX-02 and BC4**

| **Strains** | **GlpK activity**  **(U/gDCW)** | **GlpX activity**  **(U/gDCW)** | **Zwf activity**  **(U/gDCW)** | **TKT activity**  **(U/gDCW)** |
| --- | --- | --- | --- | --- |
| WX-02 | 2033.19±68.75 | 999.22±121.67 | 845.78±94.43 | 1659.43±92.08 |
| BC4 | 2411.87±47.81 | 1658.61±311.14 | 1042.23±123.21 | 2804.09±77.06 |

GlpK, GlpX, Zwf and TKT activity was measured in cell-free extracts. Data are presented as mean ± SDs of three replicates.
