# Supplementary material for: Rewiring glycerol metabolism for enhanced production of poly-γ-glutamic acid in Bacillus licheniformis
Source: Biotechnol Biofuels. 2018 Nov 9;11:306. doi: 10.1186/s13068-018-1311-9 (PMC6225680; doi:10.1186/s13068-018-1311-9)
Supplement: Supplementary file 3 — Additional file 3: Table S3. Comparison of cost with pure glycerol or crude glycerol as fermentation feedstock. [file 13068_2018_1311_MOESM3_ESM.docx]

**Table S3** **Comparison of cost with pure glycerol or crude glycerol as fermentation feedstock^a^**

| Materials | Cost ($/t) | Concentration (g/L) | Cost($/kg γ-PGA) | Cost saving |
| --- | --- | --- | --- | --- |
| Pure glycerol | 875 | 60 | 3.94 |  |
| CG_base_ | 69 | 90 | 1.64 | 58.4% |
| CG_acid_ | 121 | 78 | 1.65 | 58.1% |
| Sodium citrate | 640 | 12 |  |  |
| NH_4_Cl | 640 | 8 |  |  |
| NaNO_3_ | 533 | 15 |  |  |

^a^:The analysis of cost based on the batch fermentation of γ-PGA in 250-mL flasks containing 50 ml medium. CG_acid_ means acid crude glycerol; CG_base_ meams alkaline crude glycerol.
