# Supplementary material for: Rewiring glycerol metabolism for enhanced production of poly-γ-glutamic acid in Bacillus licheniformis
Source: Biotechnol Biofuels. 2018 Nov 9;11:306. doi: 10.1186/s13068-018-1311-9 (PMC6225680; doi:10.1186/s13068-018-1311-9)
Supplement: Supplementary file 4 — Additional file 4: Table S4. Primers used for PCR and qRT-PCR in this study. [file 13068_2018_1311_MOESM4_ESM.docx]

**Table S4 Primers used for PCR and qRT-PCR in this study**

| \| **Primer name** \| **Sequence of primer (5' to 3')^a^** \| \| --- \| --- \| \| T2-F \| ATGTGATAACTCGGCGTA \| \| T2-R \| GCAAGCAGCAGATTACGC \| \| Δ*t_glpFK_*-AF \| CGC**GGATCC**TGCTGCTTGACATCCATCTTG \| \| Δ*t_glpFK_*-AR \| TCACCTTCTCCACTGTGTCTGGTCTCCGTCACAATGTATT \| \| Δ*t_glpFK_*-BF \| AATACATTGTGACGGAGACCAGACACAGTGGAGAAGGTGA \| \| Δ*t_glpFK_*-BR \| GC**TCTAGA**GTTGTTCCGCCGAGCGAGAT \| \| Δ*t_glpFK_*-YF \| GCGGTACGCAATATGAAGC \| \| Δ*t_glpFK_*-YR \| CTGCAAATGAACCGCCAAGCG \| \| *xkdA*-AF \| CGC**GGATCC**TCTATGTTAGGCGGCAGACT \| \| *xkdA*-AR \| CGAAAACATACCACCTATCATACGAGACACCCTTTCGAAC \| \| *xkdA*-P43-F \| GTTCGAAAGGGTGTCTCGTATGATAGGTGGTATGTTTTCG \| \| P43-*glpK*-TamyL-F \| GAGAGGAATGTACACATGAAATGGAAAAGTACATTTTGTC \|  \| \| P43-*glpK*-TamyL-R \| AATCCGTCCTCTCTGCTCTTTTATTTAAAAGCCCTAGCTG \| \| P43-*glpP*-TamyL-F \| GAGAGGAATGTACACATGAAATGAGTTTTCACGATCAAAA \| \| P43-*glpP*-TamyL-R \| AATCCGTCCTCTCTGCTCTTTTATTAGTTCCAGTATTTTTTCC \| \| *xkdA*-TamyL-R \| GAGATAAATAAAACTTCAATTATTTAAAAGCCCTAGCTG \| \| *xkdA*-BF \| CAGCTAGGGCTTTTAAATAATTGAAGTTTTATTTATCTC \| \| *xkdA*-BR \| GC**TCTAGA**TTATTTTCTCAGCATATAAG \| \| *xkdA*-YF \| GAGATAATCATGCCGAA \| \| *xkdA*-YR \| ACGAAATAGAAGGGACCG \| \| *xkdG*-AF \| CGC**GGATCC**CGTCGGAGAAGTCGTGGAGTC \| \| *xkdG*-AR \| CGAAAACATACCACCTATCATTAAAGCAATCCGCTCCAAA \|  \| \| \| *xkdG*-P43-F \| TTTGGAGCGGATTGCTTTAATGATAGGTGGTATGTTTTCG \| \| P43-*glpX*-T*amyL*-F \| GAGAGGAATGTACACATGAAATGGAAAGAAGCTTATCGATG \| \| P43-*glpX*-T*amyL*-R \| AATCCGTCCTCTCTGCTCTTTTATGGCCGGATGACTAGGTTA \| \| P43-*fba*-TamyL-3 \| GAGAGGAATGTACACATGAAGTGGCTTTCGTTTCCATGAA \| \| P43-*fba*-TamyL-4 \| AATCCGTCCTCTCTGCTCTTTTATTATATTTCTATCTTTTGG \| \| P43-*pgi*-TamyL-3 \| GAGAGGAATGTACACATGAAATGACGCATGTCCGTTTTGA \| \| P43-*pgi*-TamyL-4 \| AATCCGTCCTCTCTGCTCTTTTATTATTGATTCAAACGCTTC \| \| *xkdG*-T*amyL*-R \| AACGGTTCGAACTCAGCATCGCAATAATGCCGTCGCACT \| \| *xkdG*-BF \| AGTGCGACGGCATTATTGCGATGCTGAGTTCGAACCGTT \| \| *xkdG-*BR \| GC**TCTAGA**TGCTTCCTCAGCCTGGTCTTC \| \| *xkdG*-YF \| TTTATGACGGCTGAAGAAATCCAA \| \| *xkdG*-YR \| TAAATAACCTGTTCGGGATCTTGC \| \| *xkdE*-AF \| CGC**GGATCC**ATCTCCGCCATTTCCAACC \| \| *xkdE*-AR \| CGAAAACATACCACCTATCACTATCTCCCCCTTTTCCTGT \| \| *xkdE*-P43-*F* \| ACAGGAAAAGGGGGAGATAGTGATAGGTGGTATGTTTTCG \| \| P43-*zwf*-T*amyL*-F \| GAGAGGAATGTACACATGAATTGAAAAAAGATCAAATGGA \| \| P43-*zwf*-T*amyL*-R \| AATCCGTCCTCTCTGCTCTTTTAAAGCGGCCACCAATGAA \| \| P43-*gndA*-TamyL-F \| GAGAGGAATGTACACATGAAATGGCAAAACAACAAATA \| \| P43-*gndA*-TamyL-R \| AATCCGTCCTCTCTGCTCTTTTATTATTTCATCCACTCAGT \| \| **Table S4(continued)** \| \| \| **Primer name** \| **Sequence of primer (5' to 3')^a^** \| \| P43-*rpe*-TamyL-F \| GAGAGGAATGTACACATGAAATGGTATATGTTGCTCCTTC \| \| P43-*rpe*-TamyL-R \| AATCCGTCCTCTCTGCTCTTTTATTACCCAAGCGCACCTTTTA \| \| *xkdE*-T*amyL*-R \| GCTCTCTCGGCACGAAATGTCGCAATAATGCCGTCGCACT \| \| *xkdE*-BF \| AGTGCGACGGCATTATTGCGACATTTCGTGCCGAGAGAGC \| \| *xkdE*-BR \| GC**TCTAGA**TCTTCCTCTCCCGTTTTTTG \| \| *xkdE*-YF \| GTTCTGCCGCAATTTGAAGTGATG \| \| *xkdE*-YR \| TCCTTGAACTTCTGATCGTTTC \| \| *xtmA*-AF \| CGC**GGATCC**ACTGTGCGGCTGATTGGTG \| \| *xtmA-*AR \| CGAAAACATACCACCTATCACGAGAGGCTTTTCGATCTGTTT \| \| *xtmA* -P43-F \| AAACAGATCGAAAAGCCTCTCGTGATAGGTGGTATGTTTTCG \| \| P43-*tkt*1-T*amyL*-F \| GAGAGGAATGTACACATGAAATGAATGAGGCGGCAAATGT \| \| P43-*tkt*1-T*amyL*-R \| AATCCGTCCTCTCTGCTCTTTTAAACGAGCTGTTCCGCC \| \| P43-*tkt*2-T*amyL*-F \| GAGAGGAATGTACACATGAAATGAAAACGATTGAATTAAAATCTG \| \| P43- *tkt*2-T*amyL*-R \| AATCCGTCCTCTCTGCTCTTTTAGCGATTTAACAGTTTTTTCACC \| \| *xtmA*-T*amyL*-R \| GGATTGACTTCTTTTATGACGCAATAATGCCGTCGCACT \| \| *xtmA*-BF \| AGTGCGACGGCATTATTGCGTCATAAAAGAAGTCAATCC \| \| *xtmA*-BR \| GC**TCTAGA**TATCACCGACGACAGCGACCCG \| \| *xtmA*-YF \| CCGTCATTGAAACGAGAAAGGG \| \| *xtmA*-YR \| TCTTCAGCGGGTTGGAAATGG \| \| P43-R \| TTCATGTGTACATTCCTCTC \| \| T*amyL*-F \| AAGAGCAGAGAGGACGGATT \| \| **qRT-PCR** \|  \| \| 16s rRNA-F \| TCAGCTCGTGTCGTGAGAT \| \| 16s rRNA-R \| CGATCCGAACTGAGAACAG \| \| *glpK*-F \| AGCGCTTGGTTCAGCCTATT \| \| *glpK*-R \| GTTGTACAGGCTCTCACGCT \| \| *glpF*-F \| TATGCGATTAACCCTGCCCG \| \| *glpF*-R \| AAACGCCTGCAAATGAACCG \| \| *glpD*-F \| CACGAAACTTGTACACGGCG \| \| *glpD*-R \| TCGTACACCCTCAGTCCGAT \| \| *glpX*-RT-F \| GTCCGCGGCATGAGAAAATC \| \| *glpX*-RT-R \| GAGCTTTGAGTGCAACAGCC \| \| *zwf*-RT-F \| TGCGAGCAGGTCATCAGAAG \| \| *zwf*-RT-R \| TTGGGTGTGGAAGACAGAGC \| \| *tkt*1-RT-F \| ACGTATCCGTTGTCAGCGTT \| \| *tkt*1-RT-R \| ATGCACCGAACTGGTCGATT \| \| *citZ*-RT-F \| AGCCCGAGTAAAGAAACG \| \| *citZ*-RT-R \| GTCGCCACTACATCATCAG \| \| *icd*-RT-F \| AGAGCTGCGATCGAGTATGC \| \| *icd*-RT-R \| CGGCGATGCTGTCTTTGATG \| \| *gltA*-RT-F \| GTGCTGAAACGGGAGTGA \| \| *gltA*-RT-R \| TCCGCCAGGTATTTATCG \| \| **Table S4 (continued)** \| \| \| **Primer name** \| **Sequence of primer (5' to 3')^a^** \| \| *rocG*-RT-F \| CGGTCTGGTTTATTTCGTG \| \| *rocG*-RT-R \| CTTCCGTTCCCGTCTTCT \| \| *alsD*-RT-F \| GATCGGTTTTGACGGTGAG \| \| *alsD*-RT-R \| ATCGGCACATACGGTTTTTC \| \| *alsS*-RT-F \| CGCCGATCGTTCACATTGTC \| \| *alsS*-RT-R \| TCAATGACAACAGGCCCCTC \| \| *gldA*-RT-F \| AAACGGTGTGAAAGCGATGC \| \| *gldA*-RT-R \| TTTCAAGAACAAGCTGCGCC \| \| *ackA*-RT-F \| GGGTACGCGTTCAGGTAACA \| \| *ackA*-RT-R \| AACGCGCTCTCTGACTTCAA \| \| *budC*-RT-F \| GGCGGTCAAGGAATTGGAGA \| \| *budC*-RT-R \| ATCAAGACCGCCAAGACGTT \| |  |
| --- | --- | --- | --- | --- | --- | --- | --- | --- | --- | --- | --- | --- | --- | --- | --- | --- | --- | --- | --- | --- | --- | --- | --- | --- | --- | --- | --- | --- | --- | --- | --- | --- | --- | --- | --- | --- | --- | --- | --- | --- | --- | --- | --- | --- | --- | --- | --- | --- | --- | --- | --- | --- | --- | --- | --- | --- | --- | --- | --- | --- | --- | --- | --- | --- | --- | --- | --- | --- | --- | --- | --- | --- | --- | --- | --- | --- | --- | --- | --- | --- | --- | --- | --- | --- | --- | --- | --- | --- | --- | --- | --- | --- | --- | --- | --- | --- | --- | --- | --- | --- | --- | --- | --- | --- | --- | --- | --- | --- | --- | --- | --- | --- | --- | --- | --- | --- | --- | --- | --- | --- | --- | --- | --- | --- | --- | --- | --- | --- | --- | --- | --- | --- | --- | --- | --- | --- | --- | --- | --- | --- | --- | --- | --- | --- | --- | --- | --- | --- | --- | --- | --- | --- | --- | --- | --- | --- | --- | --- | --- | --- | --- | --- | --- | --- | --- | --- | --- | --- | --- | --- | --- | --- | --- | --- | --- | --- | --- | --- | --- | --- | --- | --- | --- | --- | --- | --- | --- | --- | --- | --- | --- | --- | --- | --- | --- | --- | --- | --- | --- | --- | --- | --- | --- | --- |
| ^a^The underlines indicates an overlap region for splicing overlap extension PCR (SOE-PCR);  Generated restriction sites highlighted in bold. | |
