# Supplementary material for: Rewiring glycerol metabolism for enhanced production of poly-γ-glutamic acid in Bacillus licheniformis
Source: Biotechnol Biofuels. 2018 Nov 9;11:306. doi: 10.1186/s13068-018-1311-9 (PMC6225680; doi:10.1186/s13068-018-1311-9)
Supplement: Supplementary file 5 — Additional file 5: Figure S1. The schematic diagram of recombination strain WX02-glpX construction. [file 13068_2018_1311_MOESM5_ESM.docx]

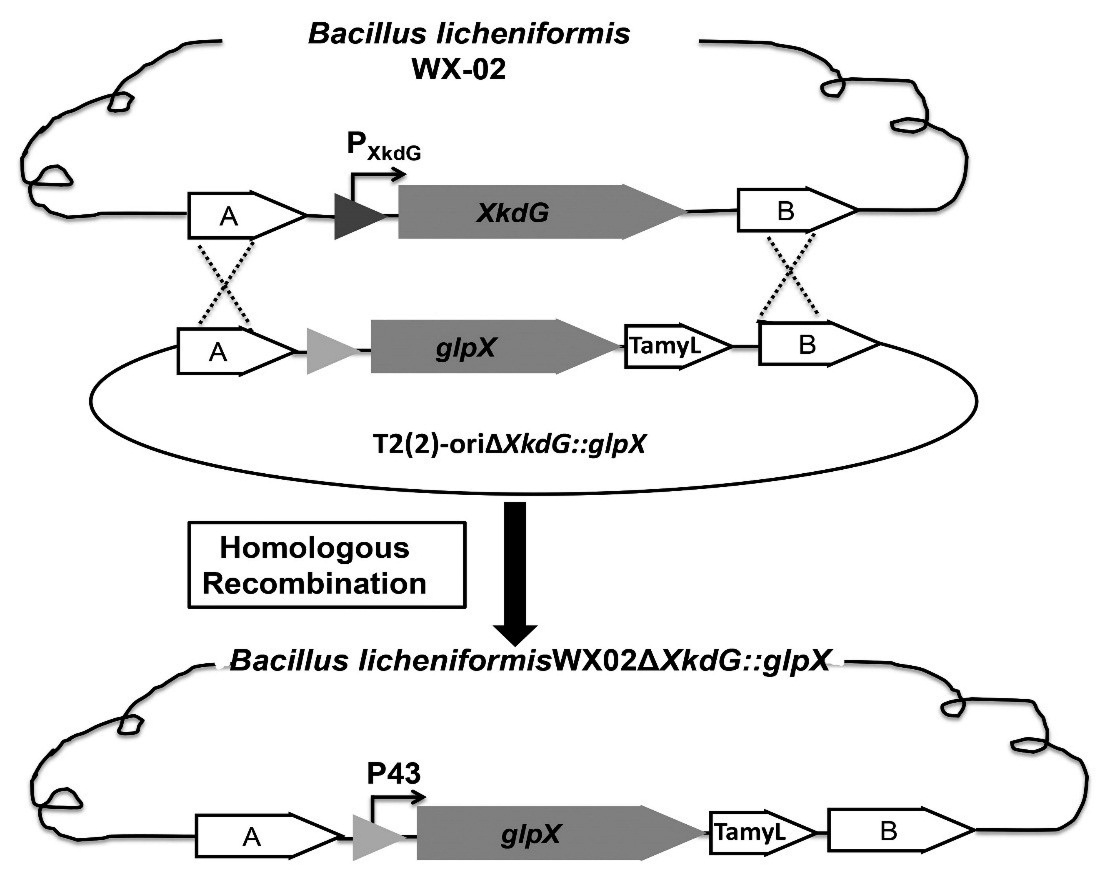


**Figure S1 Schematic diagram showing the construction of recombination strains WX02-*glpX.*** Upper panel schematic representation of the genomic organization of *xkdG* gene coding for phage capsid protein in *Bacillus licheniformis* WX-02. Lower panel homologous recombination with the plasmid T_2_-G*glpX* replaces the *xkdG*, leads to the over-expression of *glpX* under the control of promoter P43.
