# Supplementary material for: Rewiring glycerol metabolism for enhanced production of poly-γ-glutamic acid in Bacillus licheniformis
Source: Biotechnol Biofuels. 2018 Nov 9;11:306. doi: 10.1186/s13068-018-1311-9 (PMC6225680; doi:10.1186/s13068-018-1311-9)
Supplement: Supplementary file 6 — Additional file 6: Figure S2. The result of PCA and OPLS-DA with metabolome data of the WX-02 and BC4 strains cultivated in the glycerol medium. [file 13068_2018_1311_MOESM6_ESM.docx]

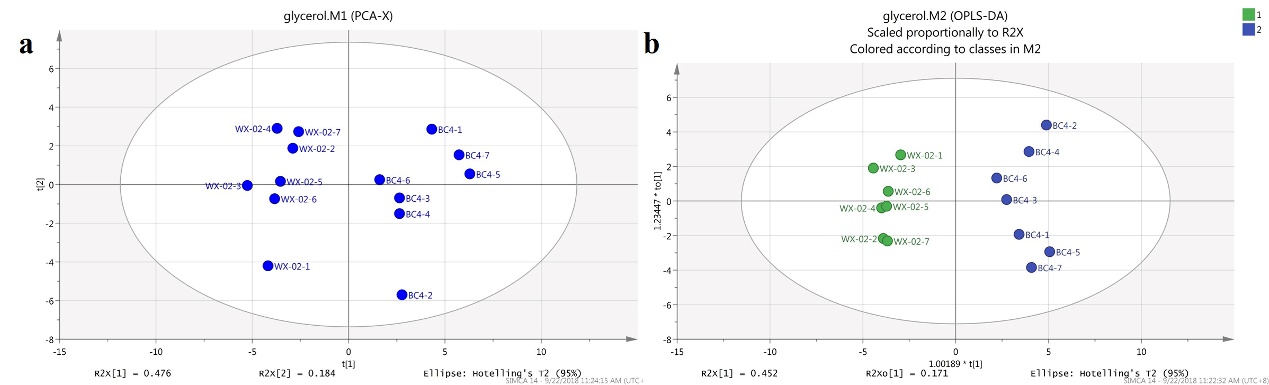


**Figure S2** The result of PCA and OPLS-DA with metabolome data of the WX-02 and BC4 strains cultivated in the glycerol medium (n=7).
